# Supplementary material for: MiR-22/GLUT1 Axis Induces Metabolic Reprogramming and Sorafenib Resistance in Hepatocellular Carcinoma
Source: Int J Mol Sci. 2025 Apr 17;26(8):3808. doi: 10.3390/ijms26083808 (PMC12027541; doi:10.3390/ijms26083808)
Supplement: Supplementary file 1 [file ijms-26-03808-s001.zip › S1_Supplementary Tables.pdf]

**Supplementary Table S1 – Characteristics of the surgical cohort (N=28) of HCC patients (Bologna cohort)**

| **Clinical Factors** | | **Frequency (%)** |
|---|---|---|
| Gender | Male/Female | 22/6 |
| Age | <65 yrs | 12/28 (42.9%) |
| Etiology | HBV | 4/28 (14.3%) |
| | HCV | 17/28 (60.7%) |
| | Alcohol abuse | 2/28 (7.1%) |
| | NASH/NAFLD | 4/28 (14.3%) |
| | None | 1/28 (3.6%) |
| Cirrhosis | | 25/28 (89.2%) |
| Serum AFP | > 20 ng/ml | 17/28 (60.7%) |
| Microvascular invasion | | 19/28 (67.9) |
| Edmondson grade | I | 0% |
| | II | 5/28 (17.9%) |
| | III | 18/28 (64.3%) |
| | IV | 5/28 (17.9%) |
| Barcelona Clinic Liver Cancer stage (BCLC) | A | 21/28 (75.0%) |
| | B | 7/28 (25.0%) |
| | C | 0% |
| | D | 0% |

**Supplementary Table S2 – Primer sequences for Real Time PCR**

| Gene | Primer sequence |
|---|---|
| **miR-22** | Fw 5’-TCGAACTCGAGCCGGTGTGTGCTGGGTGC-3’<br>Rv 5’-TCGACTCGAGCTTGCCCAAAACGTATCATCCACC-3’ |
| **ß-ACTIN** | Fw 5’-ACCTTCTACAATGAGCTGCG-3’<br>Rv 5’-CCTGGATAGCAACGTACATGG-3’ |
| **GAPDH** | Fw 5’-ACATCGCTCAGACACCATG-3’<br>Rv 5’-TGTAGTTGAGGTCAATGAAGGG-3’ |
| **HIF1A** | Fw 5’-AACATAAAGTCTGCAACATGGAAG-3’<br>Rv 5’-TTTGATGGGTGAGGAATGGG-3’ |
| **ALDOA** | Fw 5’-GGTGTCATCCTCTTCCATGAG-3’<br>Rv 5’-GTAGTCTCGCCATTTGTCCC-3’ |
| **GLUT1** | Fw 5’-AAAGTGACAAGACACCCGAG-3’<br>Rv 5’-TGTCAGGTTTGGAAGTCTCATC-3’ |
| **PFK2** | Fw 5’-GGCAAGACCTACATCTCCAAG-3’<br>Rv 5’-ATGGCTTCCTCATTGTCGG-3’ |
| **LDHA** | Fw 5’-CGTCAGCAAGAGGGAGAAAG-3’<br>Rv 5’-GCCACGTAGGTCAAGATATCC-3’ |
| **SOX2** | Fw 5’-CACACTGCCCCTCTCAC-3’<br>Rv 5’-TCCATGCTGTTTCTTACTCTCC-3’ |
| **PROM1** | Fw 5’-GTGGATGCAGAACTTGACAAC-3’<br>Rv 5’-ACCCTTTTGATACCTGCTACG-3’ |
| **SNAI1** | Fw 5’-ATCCAGAGTTTACCTTCCAGCAG-3’<br>Rv 5’-CCAGGACAGAGTCCCAGATG-3’ |
| **CASP3** | Fw 5’-ACTGGACTGTGGCATTGAG-3’<br>Rv 5’-GAGCCATCCTTTGAATTTCGC-3’ |
| **FASN** | Fw 5’-CAAGCTGAAGGACCTGTCTAG-3’<br>Rv 5’-CGGAGTGAATCTGGGTTGATG-3’ |
| **ACLY** | Fw 5’-AGACTATCCTCTCCCTCATGAC-3’<br>Rv 5’-CGTTGGTGAAGTTTGCGATG-3’ |
| **ANGPT2** | Fw 5’-ATGCAGTACAGAACCAGACG-3’<br>Rv 5’-AAGTTCAAGTCTCGTGGTCTG-3’ |
| **PKM2** | Fw 5’-AAGTGTGACGAGAACATCCTG-3’<br>Rv 5’-ACCATTTTCCACCTCCGTC-3’ |
| **PFK1** | Fw 5’-TGACCAAAGATGTGACCAAGG-3’<br>Rv 5’-GCGAACCACTCTTAGATACCG-3’ |

| | |
|---|---|
| **HK2** | Fw 5’-GGGACAATGGATGCCTAGATG-3’<br>Rv 5’-GTTACGGACAATCTCACCCAG-3’ |
| **G6PD** | Fw 5’-AGAACATTCACGAGTCCTGC-3’<br>Rv 5’-GTGGTCGATGCGGTAGATC-3’ |
| **PGD** | Fw 5’-TGGCTGGACTCTCAATTATGG-3’<br>Rv 5’-GGGTTTCGATCAAATGCATCC-3’ |
| **CD105** | Fw 5’-ATAGGACTGTCTTCATGCGC-3’<br>Rv 5’-GTAGATGTACCAGAGTGCAGC-3’ |
| **Rat_AFP** | Fw 5’- AAAGACCAGGATCAGGAAGC-3’<br>Rv 5’- CCATCAAACCGAAAAGCTCAC-3’ |
| **Rat_GLUT1** | Fw 5’- TGATTGGTTCCTTCTCTGTGG-3’<br>Rv 5’- CCCAGGATCAGCATCTCAAAG-3’ |
| **Rat_CASP3** | Fw 5’- ATCAAAGCTTAGTGTCCTGAGG-3’<br>Rv 5’- TCCATCGACTTGCTTCCATG-3’ |
| **Rat_PUMA** | Fw 5’- AGAAATGGAGCCCAACTAGTG-3’<br>Rv 5’- GGCAGTCCAGTATGCTACATG-3’ |
| **Rat_BMF** | Fw 5’- GAGGTACAGATCGCCAGAAAG-3’<br>Rv 5’- TCCCTGTTTTCTCGTCTGTTC-3’ |
| **Rat_ß-ACTIN** | Fw 5’-CACTTTCTACAATGAGCTGCG-3’<br>Rv 5’-CTGGATGGCTACGTACATGG-3’ |
| **HIF1A.13.3 DsiRNA C** | Fw 5’-CGGUUGAAUCUUCAGAUAUGAAAAT-3’<br>Rv 5’-AUUUUCAUAUCUGAAGAUUAACCGGU-3’ |
| **GLUT1_MUT** | Fw 5’- CAGGAGCACAaagtGCTGGATGAGAC-3’<br>Rv 5’-AGAGATCCTTAGGGC-3’ |
| **GLUT1_seq** | Fw 5’-GATTCCCAAGTGTGAGTCGC-3’<br>Rv 5’-GACATCATTGCTGGCTGGAG-3’ |

**Supplementary Table S3 - Antibodies for WB analysis**

| Antibody | Catalogue number | Company |
|---|---|---|
| **Cleaved caspase-3 (Asp175)** | #9661 | Cell Signaling Technologies |
| **BAX** | #2772 | Cell Signaling Technologies |
| **GAPDH** | #2118 | Cell Signaling Technologies |
| **HIF-1** | #E-AB-31662 | ElabScience |
| **Lamin B** | #sc-6216 | Santa Cruz Biotechnology |
| **SNAIL** | #3879 | Cell Signaling Technologies |
| **GLUT1** | #73015 | Cell Signaling Technologies |
| **phospo-AKT (Ser473) (D9E) XP** | #4060 | Cell Signaling Technologies |
| **CD133 (D4W4N)** | #86781 | Cell Signaling Technologies |
| **SOX2 (D9B8N)** | #23064 | Cell Signaling Technologies |
