# Supplementary material for: MiR-22/GLUT1 Axis Induces Metabolic Reprogramming and Sorafenib Resistance in Hepatocellular Carcinoma
Source: Int J Mol Sci. 2025 Apr 17;26(8):3808. doi: 10.3390/ijms26083808 (PMC12027541; doi:10.3390/ijms26083808)
Supplement: Supplementary file 1 [file ijms-26-03808-s001.zip › S2_supplementary figure legends.pdf]

Supplementary Figure S1. High miR-22 expression associates with metabolic changes and cell cycle arrest. (**A**) Volcano plot of deregulated genes in high versus low miR-22-expressing HCCs from TCGA cohort. (**B**) Gene Set Enrichment Analysis (GSEA) of high versus low miR-22-expressing HCCs from TCGA cohort.

Supplementary Figure S2. MiR-22 regulates 3D cell growth, stemness properties and HIF-1A pathway in HCC. (**A**) Real Time PCR analysis of miR-22 expression in HCC cell lines, as well as in tumor and surrounding liver (SL) tissues in HCC patients from the Bologna cohort. Y-axis reports $2^{-\Delta\Delta Ct}$ values corresponding to miR-22 expression. Mean ± SD values are displayed. U6RNA was used as housekeeping gene. Real Time PCR analysis was performed in triplicate. (**B**) Histogram showing the number of miR-22-silenced (MZIP-22) or control (shRNA) HepG2 spheroids. Normalized mean ± SD values are displayed. The analysis was performed in two independent experiments in triplicate. (**C**) Growth curves and representative images of miR-22-silenced (MZIP-22) or control (shRNA) HepG2 monospheroids. Y-axis reports monospheroids volume normalized on T0. Mean ± SD values are displayed. The analysis was performed in two independent experiments in quadruplicate. (**D**) Box plot graph of SOX2 expression in high versus low miR-22 expressing HCCs from TCGA cohort. (**E**) Kaplan-Meier curves of high and low SOX2 expressing HCCs from TCGA cohort. (**F**) Box plot graph of HIF1A expression in high versus low miR-22 expressing HCCs from TCGA cohort. (**G**) Kaplan-Meier curves of high and low HIF1A expressing HCCs from TCGA cohort. (**H**) Real Time PCR analysis of GLUT1 and ALDOA expression in miR-22-silenced (MZIP-22) HepG2 or miR-22-overexpressing (pMXs-22) Huh-7 spheroid and related controls (shRNA). Y-axes report $2^{-\Delta\Delta Ct}$ values corresponding to normalized gene expression. Mean ± SD values are displayed. Real Time PCR was performed in two independent experiments in triplicate. WB analysis of HIF-1A expression in the same spheroids. GAPDH was used as housekeeping gene in both

analyses. WB analysis was performed in two independent experiments. (**I**) Box plot graph of GLUT1, ALDOA, PFK2 expression in high versus low miR-22 expressing HCCs from TCGA cohort.

Supplementary Figure S3. MiR-22 regulates EMT and tumorigenesis in preclinical models. (**A**) Representative images (10X magnification) of control (shRNA) and miR-22-silenced (MZIP-22) HepG2 cells, showing different morphology. Scale bars: 300 µm. (**B**) Box plot graphs representing tumor volume of miR-22-silenced (MZIP-22) HepG2 cells and miR-22-overexpressing (pMXs-22) Huh-7 cells and related controls (shRNA) in xenograft mice (N=5 per group) at sacrifice. Y-axes report the tumor volume (mm^3) measured by caliper (1/2 $D*d^2$).

Supplementary Figure S4. GLUT1 is a hypothetic target of miR-22 and regulates metabolic shift in HCC. (**A**) Complementary miR-22 (miR-22-3p) binding site in GLUT1-3'UTR (SLC2A1 3'UTR), as displayed by TargetScan. Stars over the seed sequence represent modified bases in the mutated vector used in the dual-luciferase reporter assay. (**B**) Real Time PCR analysis of HIF1A and GLUT1 expression in control (shRNA) HepG2 cells following HIF1A silencing (left graph). GLUT1 expression in control and miR-22-silenced (MZIP-22) HepG2 cells following HIF1A silencing at 24 and 48 hours (right graph). Y-axes report $2^{-\Delta\Delta Ct}$ values corresponding to normalized gene expression. Mean ± SD values are displayed. GAPDH was used as housekeeping gene. Real Time PCR was performed in two independent experiments in triplicate. Scramble: negative control oligonucleotides. (**C**) Real Time PCR analysis of GLUT1 expression levels in HepG2 or Huh-7 cells in different pH conditions. Y-axes report $2^{-\Delta\Delta Ct}$ values corresponding to GLUT1 expression. Mean ± SD values are displayed. GAPDH was used as housekeeping gene. Real Time PCR was performed in two independent experiments in triplicate. (**D**) Enzymatic assay measuring glucose uptake and HPLC analysis measuring extracellular lactate in control (shRNA) and miR-22-overepressing Huh-7 cells (pMXs-22) at different time points (24, 48, 72 hours). Mean ± SD values are displayed. Two independent experiments were analyzed in triplicate. NMR analysis of intracellular lactate levels in

the same cells. Y-axis reports peak intensity normalized to control. Mean ± SD values are displayed. NMR analysis was performed in three biological replicates. (**E**) Oxygen consumption rate in miR-22-silenced (MZIP-22) HepG2 cells and miR-22-overexpressing (pMXs-22) Huh-7 cells and related controls (shRNA) measured in standard medium (endogenous respiration), in the presence of oligomycin A (oligo) and carbonyl cyanide 4-(trifuoromethoxy) phenylhydrazone (FCCP). Mean ± SD values are displayed. Three independent experiments were performed. (**F**) Enzymatic activity of citrate synthase (CS) in in miR-22-silenced (MZIP-22) HepG2 cells and miR-22-overexpressing (pMXs-22) Huh-7 cells and related controls (shRNA). Y-axes report the enzymatic activity (μmol*min^{-1}*mg^{-1}) normalized to control. Mean±SD values are displayed. Three independent experiments were performed. (**G**) Real Time PCR analysis of glycolytic enzymes (PFK1, HK2, PKM2) expression in in miR-22-silenced (MZIP-22) HepG2 cells and miR-22-overexpressing (pMXs-22) Huh-7 cells and related controls (shRNA). Y-axes report $2^{-\Delta\Delta Ct}$ values corresponding to normalized genes expression. Mean ± SD values are displayed. GAPDH was used as housekeeping gene. Real Time PCR was performed in two independent experiments in triplicate. Box plot graph of PKM expression in high versus low miR-22 expressing HCCs from TCGA cohort. (**H**) Mass spectrometry (LC-MS) analysis of fructose 1,6-bisphosphate (F-1,6-BP) in miR-22-silenced (MZIP-22) HepG2 cells and miR-22-overexpressing (pMXs-22) Huh-7 cells and related controls (shRNA). LC-MS analysis was performed in duplicate in three biological replicates. (**I**) Representative images (20X magnification) and related histograms of PAS staining in xenograft mice (N=5 per group) obtained by injection of in miR-22-silenced (MZIP-22) HepG2 cells and miR-22-overexpressing (pMXs-22) Huh-7 cells and related controls (shRNA). Scale bars: 100 μm. Y-axes reports the percentage of PAS positive cell area normalized to control. Mean ± SD values are displayed. Five randomly selected fields were analyzed per each tumor mass.

Supplementary Figure S5. MiR-22 modulates oxidative stress in HCC cells. (**A**) Intracellular H_2O_2 levels in miR-22-silenced (MZIP-22) HepG2 cells and miR-22-overexpressing (pMXs-22)

Huh-7 cells and related controls (shRNA). Normalized mean ± SD values are displayed. Two independent experiments were run in quadruplicate. (**B**) Real Time PCR analysis of HAO2 expression in miR-22-silenced (MZIP-22) HepG2 cells and miR-22-overexpressing (pMXs-22) Huh-7 cells and related controls (shRNA). Y-axes report $2^{-\Delta\Delta Ct}$ values corresponding to normalized gene expression. Mean ± SD values are displayed. GAPDH was used as housekeeping gene. Real Time PCR was performed in two independent experiments in triplicate. (**C**) Measurement of mitochondrial ROS levels with the fluorogenic dye MitoSOX in miR-22-silenced (MZIP-22) HepG2 cells and miR-22-overexpressing (pMXs-22) Huh-7 cells and related controls (shRNA). Mean ± SD values are displayed. Y axes report fluorescent emission normalized to controls. The analysis was performed in two independent experiments in triplicate. (**D**) Measurement of lipid peroxidation levels with fluorogenic dye BODIPY® 581/591 in miR-22-silenced (MZIP-22) HepG2 cells and miR-22-overexpressing (pMXs-22) Huh-7 cells and related controls (shRNA). Mean ± SD values are displayed. Y axes report fluorescent emission normalized to controls. The analysis was performed in two independent experiments in triplicate. (**E**) Glutathione (GSH) levels in miR-22-silenced (MZIP-22) HepG2 cells and miR-22-overexpressing (pMXs-22) Huh-7 cells and related controls (shRNA). Normalized mean ± SD values are displayed. Two independent experiments were run in quadruplicate. (**F**) Real Time PCR analysis of G6PD and PGD expression in miR-22-silenced (MZIP-22) HepG2 cells and miR-22-overexpressing (pMXs-22) Huh-7 cells and related controls (shRNA). Y-axes report $2^{-\Delta\Delta Ct}$ values corresponding to normalized gene expression. Mean ± SD values are displayed. GAPDH was used as housekeeping gene. Real Time PCR was performed in two independent experiments in triplicate. (**G**) Mass spectrometry (LC-MS) analysis of fructose 6-phosphogluconate (6PG) in miR-22-silenced (MZIP-22) HepG2 cells and miR-22-overexpressing (pMXs-22) Huh-7 cells and related controls (shRNA). LC-MS analysis was performed in duplicate in three biological replicates. (**H**) Real Time PCR analysis of GSTA4 and NRF2 expression in miR-22-silenced (MZIP-22) HepG2 cells and miR-22-overexpressing (pMXs-22) Huh-7 cells and related controls (shRNA). Y-axes report $2^{-\Delta\Delta Ct}$ values corresponding to normalized gene expression. Mean ±

SD values are displayed. GAPDH was used as housekeeping gene. Real Time PCR was performed in two independent experiments in triplicate. (**I**) Box plot graph of G6PD expression in high versus low miR-22 expressing HCCs from TCGA cohort.

Supplementary Figure S6. MiR-22/GLUT1 axis modulates sorafenib response and angiogenesis in preclinical models of HCC. (**A**) Real Time PCR analysis of miR-22 and GLUT1 expression in sorafenib-resistant (SR) HepG2 cells. Y-axes report $2^{-\Delta\Delta Ct}$ values corresponding to normalized expression. Mean ± SD values are displayed. RNU6B and GAPDH were used as housekeeping genes. The Real Time PCR was performed in two independent experiments in triplicate. (**B**) Cell viability and caspase assays of in miR-22-silenced (MZIP-22) HepG2 cells and miR-22-overexpressing (pMXs-22) Huh-7 cells and related controls (shRNA) following sorafenib treatment (5 µM, 48 h). Y-axes report chemiluminescent signals normalized to controls. Mean ± SD values are displayed. Two independent experiments were performed in quadruplicate. WB analysis of pAKT and apoptotic markers in the same setting. WB was performed in two independent experiments, and GAPDH was used as housekeeping gene. (**C**) Box plot graphs of miR-22 and GLUT1 expression in tumor nodules (HCC) and surrounding livers (SL) from DEN-HCC rats treated with sorafenib (N=15). Y-axes report $2^{-\Delta\Delta Ct}$ values corresponding to miR-22 or GLUT1 normalized expression. Mean ± SD values are displayed. RNU6B and GAPDH were used as housekeeping genes. Real Time PCR was run in triplicate. (**D**) Representative images of CD31 staining (20X or 40X magnification; scale bars: 100 µm or 50 µm) and related histograms in xenograft mice (N=5 per group) obtained after injection of miR-22-silenced (MZIP-22) and control (shRNA) HepG2 cells. Y-axes report the number of CD31-positive vasculature per field. Mean ± SD values are displayed. Five randomly selected fields were analyzed per each tumor. Real Time PCR analysis of ANGPT2 expression in the same animals. Y-axis reports $2^{-\Delta\Delta Ct}$ values corresponding to normalized gene expression. Mean ± SD values are displayed. GAPDH was used as housekeeping gene. Real Time PCR was performed in triplicate. (**E**)

Real Time PCR analysis of ANGPT2 expression levels in HCC nodules and surrounding livers (SL) of patients from the Bologna cohort. Y-axis reports $2^{-\Delta\Delta Ct}$ values corresponding to gene expression. Mean ± SD values are displayed. GAPDH was used as housekeeping gene. The Real Time PCR was run in triplicate. (**F**) Real Time PCR analysis of extracellular miR-22 expression levels in supernatants from miR-22-overexpressing (pMXs-22) and control (shRNA) Huh-7 cells. Y-axis reports $2^{-\Delta\Delta Ct}$ values corresponding to normalized miR-22 levels. Mean ± SD values are displayed. Cel-39 was used as housekeeping gene. The Real Time PCR was performed in two independent experiments in triplicate. (**G**) Representative images of microtubules formation assay of HUVEC cells after exposure to supernatant of miR-22-overexpressing (pMXs-22) and control (shRNA) Huh-7 cells. Tube formation quantification is shown in the top right graph. Y-axis reports the number of nodes. CD105 expression in HUVEC cells is shown in the bottom right graph. Y-axis reports normalized $2^{-\Delta\Delta Ct}$ values. Mean ± SD values are displayed. GAPDH was used as housekeeping gene. The microtubule formation assay was performed in two independent experiments in quadruplicate, whereas the Real Time PCR was performed in triplicate. (**H**) Box plot graph of VEGF and ANGPT2 expression in high versus low miR-22 expressing HCCs from TCGA cohort.
