# Supplementary material for: MiR-22/GLUT1 Axis Induces Metabolic Reprogramming and Sorafenib Resistance in Hepatocellular Carcinoma
Source: Int J Mol Sci. 2025 Apr 17;26(8):3808. doi: 10.3390/ijms26083808 (PMC12027541; doi:10.3390/ijms26083808)
Supplement: Supplementary file 1 [file ijms-26-03808-s001.zip › S3_supplementary material.pdf]

TCGA Dataset Analysis

Data from The Cancer Genome Atlas Liver Hepatocellular Carcinoma project (TCGA-LIHC) cohort including 374 HCC and 50 non-tumor liver samples was retrieved from the GDC portal. Tumoral samples were divided based on miR-22 expression levels into high and low expressing groups. Differential expression analysis was performed in R software v. 4.2.1. (R Core Team, 2020) using the DeSeq2 package (Love et al., 2014) with the low expressing group set as a reference. The Benjamini-Hochberg method was used to adjust the p-value for multiple comparisons. Gene set enrichment analysis (GSEA) was done to further explore the roles of the resulting deregulated genes (Korotkevich et al., 2021) using the Hallmarks, Gene Ontology and KEGG Human Molecular Signatures Databases (MSigDB) (Subramanian et al., 2005; Mootha et al., 2003). Genes of interest were plotted using boxplots.

Cytoplasmic Protein Extraction

One to five million cells were collected by centrifugation, and proteins extracted according to manufacturer's instructions. Briefly, cell pellets were suspended in CER I reagent and protease inhibitor cocktail for 10 minutes. Then, CER II reagent was added (5 min incubation), and tubes were centrifuged at maximum speed for 5 min. The supernatant represented the cytoplasmic fraction. The remaining pellets were suspended in NER reagent and protease inhibitor cocktail (40 min incubation) and centrifuged at maximum speed for 10 min to obtain is the nuclear extract. All steps were performed at 4°C. The volume ratio of CER I:CER II:NER reagents was maintained at 200:11:100. The purity of cytoplasmic and nuclear fractions was determined using GAPDH and lamin-B, respectively.

Monospheroids formation assays

Monospheroids protocol is briefly detailed. Agarose was dissolved in PBS to obtain a 3% solution, then diluted 1:2 in warm culture media. 50 µl of agarose were added to each well of a flat-bottomed 96-well cell culture plate and leaved to cool under the hood. Cells were seeded in a final volume of 190 µl in the appropriate culture medium (350 cells/well for HepG2 cell line, 2000 cells/well for Huh-7 cell line). The plate was centrifuged for 10 min at 1500 rpm to collect the cells in the center of the well and placed in the incubator until spheroid formation. Images were taken using the Incucyte Live Imaging System (Sartorious) and analyzed with the Spheroid Analysis Software Module.

Sorafenib resistant HCC cells

To obtain sorafenib-resistant (SR) HepG2 cell clone, HepG2 cells were treated with increasing doses of sorafenib, starting from 1 μM. Cells were incubated with the designated concentration of sorafenib in MEM medium supplemented with 10% FBS, 1% L-glutamine and 1% penicillin–streptomycin and incubated at 37 °C in 5% CO2. Viable cells were subcultured every week, increasing sorafenib concentration by 0.25 μM up to a final concentration of 4 μM.

PAS staining

Four-micrometer thick FFPE sections were processed for Periodic Acid Schiff (PAS) staining. Briefly, oxidation was obtained by incubating slides in 0.5% Periodic Acid solution for 5 minutes whereas staining was performed by adding Schiff's reagent for 7 minutes. Counterstaining was obtained with Meyer's hematoxylin. Cells were seeded onto a coverslip in a 6-well plate at $1x10^5$ cells/well for Huh-7 cells or 3 $x10^5$ cells/well for HepG2 cells. After washing with PBS, cells were fixed in 4% paraformaldehyde at RT for 1 hour. Samples were incubated for 10 minutes at RT with Periodic Acid solution, treated with Shiff's reagent for 20 minutes at RT in the dark and counterstained with hematoxylin. The coverslips were washed with tap water for 5 minutes, let dry for 10 minutes, and then fixed in glycerol based mounting medium on a microscope slide. Images were acquired using a DM750 microscope with ICC50 digital camera (Leica). At least 10 randomly chosen fields (20X magnification) were acquired. To evaluate the PAS signals from images, we subjected the background-corrected images to the commonly used color deconvolution with defined color vectors, and then percentage of cell area staining positive for PAS was determined using ImageJ Software tools (ImageJ, National Institutes of Health). The experiment was repeated twice in triplicate.

Oxygen consumption determination

The oxygen consumption in miR-22-overexpressing HepG2 cells and miR-22 silenced Huh-7 cells and controls was measured using a thermostatically controlled oxygraph chamber at 37°C equipped with Clark electrode (Yellow Springs Instrument YSI 53). Briefly, 2.5-5.0 x 10^5 cells were seeded in T25 cell culture flasks and grown for 48 hours in standard culture conditions. Then, cells were washed in NaCl 0,9%, trypsinized, and resuspended in complete medium. Endogenous respiration was measured in complete medium (basal respiration), after the addition of 1 μM of the ATPase inhibitor oligomycin A (non-phosphorylating respiration), and of 500 nM of the uncoupler carbonyl cyanide 4-(trifluoromethoxy) phenylhydrazone (FCCP) (maximal respiration). The ATP-linked respiration was calculated by subtracting non-phosphorylating to basal respiration. All data were expressed as

nmoles $O_2 \cdot min^{-1} \cdot mg\ proteins^{-1}$. Protein concentration was determined by the Lowry method. Four biological replicates were performed (Liparulo et al., 2021).

Citrate synthase activity

The citrate synthase (CS) activities were measured using a UV-vis spectrophotometer (V-750, JASCO) equipped with a cuvette stirring device and thermostatic control. Cells were collected by centrifugation, washed with PBS, and suspended in 20 mM hypotonic potassium phosphate buffer (pH 7.5) followed by three cycles of freeze-thawing. CS activity was followed at λ= 412nm in 100 mM TRIS buffer (pH 8), 0.1% Triton, 0.1 mM acetyl-coA, 0.5 mM oxalacetate and 0.1 mM 5,5'-dithiobis-2-nitrobenzoic acid (DTNB, ε= 13.6 mmol −1 cm −1) and 30 µg of cell lysate at 30°C. Data were normalized to protein content determined by the Lowry method. Four biological replicates were performed.

Extracellular lactate determination

The lactate amount was assessed by HPLC. Cells were seeded in 6-well plates at 0.9 $x10^5$ cells/well for Huh-7 cells or 1.8 $x10^5$ cells/well for HepG2 cells and grown for 24-72 hours in standard culture conditions. The culture medium was collected and diluted 1:5 in the mobile phase consisting of 50 mM KH2PO4, pH 2.4 and centrifuged at 14000 g for 5 min at 4 °C. The supernatant was collected and injected in an HPLC system (Agilent 1100 Series System) equipped with a phenylic column (Agilent ZORBAX SB-Phenyl, 5 µm, 250 × 4.6 mm), using a mobile phase consisting of 50 mM KH2PO4, pH 2.4, at a flow rate of 0.8 mL·min−1. Absorbance at λ 210 nm was monitored by a photodiode array detector. Lactate quantification was obtained by peak area measurement compared with standard curves and normalized on cell number. The analysis was performed in triplicate.

Lipid quantification assay

The intracellular lipid droplets were visualized by staining the cells with the Nile Red fluorescent probe (Thermo Fisher Scientific). Briefly, cells were seeded onto a coverslip placed in a 6-well plate at a density of 0.9 $x10^5$ cells/well for Huh-7 cells or 1.8 $x10^5$ cells/well for HepG2 cells. Subsequently, cells were washed and fixed with 4% paraformaldehyde for 1 hour at RT. Cells were rinsed twice with glycine 50 mM in PBS. Lipid droplets were stained with 1 ng/ml of Nile Red in PBS for 10 minutes in the dark. Cells were washed with bidistilled water and coverslips mounted in a glycerol based mounting medium on a microscope slide. Images were acquired using a Nikon C1si confocal microscope; 30 randomly chosen fields for each condition were analyzed by ImageJ software standard

tool (ImageJ, National Institutes of Health) (Rizzardi et al., 2021). The experiment was repeated twice in triplicate.

Radical Oxygen Species and mitochondrial anion superoxide measurement

The mitochondrial anion superoxide production was determined by using MitoSOX™ Red (Molecular Probes). Briefly, cells were seeded as above in 96-well plates (OptiPlate Black; PerkinElmer). Cells were incubated with 5 μM of MitoSOX™ Red for 30 minutes in complete medium. After this time, cells were washed with HBSS, and the fluorescence emission was measured (λex= 510 nm; λem= 580 nm) with a multiplate reader (Liparulo et al., 2021). For both experiments, fluorescence emission was normalized on protein content determined by Lowry's assay. The experiment was repeated twice in triplicate.

Glutathione levels analysis

Glutathione levels were assessed using the GSH/GSSG-GloTM kit (Promega) following manufacturer's instructions. Luminescence was determined using a multiplate reader (Spark; Tecan). The experiment was repeated twice in triplicate.

Hydrogen peroxide levels analysis

Hydrogen peroxide levels were assessed using the ROS-GLO^{TM} H_2O_2 Assay kit (Promega) following manufacturer's instructions. Luminescence was determined using a multiplate reader (Spark; Tecan). The experiment was repeated twice in triplicate.

Lipid Peroxidation Assay

The assessment of cell membrane peroxidation was performed using the lipid peroxidation sensor BODIPY® 581/591 (Thermo Fisher Scientific), as described by Rizzardi *et al*. (Rizzardi et al., 2021). Upon oxidation of the polyunsaturated butadienyl portion of the dye, a shift in the fluorescence emission peak is observed, moving from approximately 590 nm (red) to 510 nm (green). Briefly, cells were seeded in a μ-Slide 8 Well (Ibidi) according to the manufacturer's instructions and stained the following day with 1 μM of BODIPY® 581/591 C11 in complete medium for 1 hour. Cells were then carefully washed twice with HBSS. Images were acquired using a Nikon C1si confocal microscope (Nikon), and fluorescence intensity was analyzed using ImageJ software. At least 50 cells per experimental condition were evaluated, and data were expressed as the ratios of green-to-red fluorescence intensities ± standard deviations.

HR-NMR analysis

For the HR-MAS experiment, cells were introduced in a 50 µl MAS zirconia rotor (4 mm OD) with 10 µl of deuterated water (D2O), closed with a cylindrical insert to increase sample homogeneity, then transferred into the probe cooled to 5 °C to prevent cell degradation processes (Righi et al. 2007). 1H and 13C HR-MAS NMR spectra were recorded with a Bruker Avance400 spectrometer operating at 400.13 and 100.61 MHz, respectively. Samples were spun at 4000 Hz. The setup of the experiments is about 20 min. Three different types of one-dimensional (1D) proton spectra were acquired by using: i) a composite pulse sequence (zgcppr) (Price et al. 1999), with 2.5 s water-presaturation during relaxation delay, 8 kHz spectral width, 32 k data points, 64 scans; ii) a water suppressed spin-echo Carr-Purcell-Meiboom-Gill (CPMG) sequence (cpmgpr), with 1.5 s water presaturation during relaxation delay, 1 ms echo time (τ), and 360 ms total spin-spin relaxation delay (2nτ), 8 kHz spectral width, 32 k data points, 128 scans; and iii) a sequence for diffusion measurements based on stimulated echo and bipolar-gradient pulses (ledbpgp2s1d) with big delta 200 ms, eddy current delay Te 5 ms, little delta 2*2 ms, sine-shaped gradient with 32 G/cm followed by a 200 µs delay for gradient recovery, 8 kHz spectral width, 8 k data points, 256 scans. Two-dimensional (2D) 1H,1H-COrrelation SpectroscopY (COSY), TOtal Correlation SpectroscopY (TOCSY) and 1H,13C Heteronuclear Single Quantum Coherence (HSQC) experiments were performed, as previously described (Schenetti 2006) are also acquired to characterize the metabolic fingerprint ef HCC cells.

Data Processing, Bioinformatics, and Statistical Analysis

The CPMG spectra highlighted the narrow signals of metabolites and attenuate the broad signals of macromolecules and lipids, and these were the spectra used for statistics. The identification of metabolites was based on literature data, on HMDB (http://www.hmdb.ca, version 5.0) and BMRB (https://bmrb.io) digital libraries and on our knowledge about the shape and position of the signals checked by 2D experiments. CPMG spectra were transformed with 1 Hz line broadening, manually phased, baseline corrected, aligned and binned (0.002 ppm) with MNova software package [MestReNova, ver. 11.4, 4-18998, 2017 Mestrelab Research S. L., Santiago de Compostela, Spain]. The chemical shift scale was calibrated using the doublet of alanine set at 1.48 ppm. Each spectrum was normalized to the total number of cells. The areas of selected signals identified were estimated by deconvolution through the MNova Line Fitting routine.

Targeted metabolomics analysis

Chemicals and Reagents

6-Phosphogluconic acid trisodium salt, D-Fructose 1,6-bisphosphate trisodium salt hydrate, 2-Deoxy-D-glucose 6-phosphate sodium salt (IS) were provided by Merck. LC-MS grade methanol, LC-MS

grade formic acid, HPLC *ter*-butil metil etere (MTBE) and HPLC grade were also provided by Merck; UPLC grade water was provided by VWR chemicals.

Instrument and Conditions

Semi-quantitative analysis was performed using a triple quadrupole turbo ion spray mass spectrometer (Sciex 4500 QTRAP, Concord) coupled with a UHPLC system (Nexera X2 UHPLC, Shimadzu Corporation) equipped with a Kinetex PFP, 100x4.6 mm I.D., 2.6 µm 100 Å column (Phenomenex). The analytical column was maintained at a temperature of 30°C during analysis. Chromatographic separation was achieved using isocratic flow of a solvent composed of 100% mobile phase A, followed by a 2.5 min wash using 100% mobile phase B. The flow rate was set at 0.8 mL/min. The mobile phases consisted of (A) 0.1% FA in water and (B) 0.1% FA in methanol. The MS/MS analyses were carried out using multiple reaction monitoring (MRM) negative ionization mode. The ion spray voltage was set at 4500 V. The curtain and collision gas (nitrogen) pressures were set at 30 PSI; the nebulizer and heater gas pressures were set respectively at 35 PSI and 50 PSI. The ion spray probe temperature was set at 550°C. The declustering potentials, entrance potentials, collision energies, and collision cell exit potential were optimized by direct infusion of pure analytical standards at a concentration of 1 µg/mL. The MS/MS parameters are listed in the table below.

Analyte name	Q1	Q3	DP	EP	CE	CXP
6-Phosphogluconic acid	275.0	96.8	-30	-5	-19	-10
D-Fructose 1,6-bisphosphate	338.7	96.9	-60	-8	-25	-8
2-Deoxy-D-glucose 6-phosphate (IS)	242.4	96.9	-70	-8	-20	-10

Stock Solutions and Standards

Analytical standards and 2-Deoxy-D-glucose 6-phosphate (IS) were dissolved in 50% LC-MS grade methanol, at a concentration of 5 mg/mL. Appropriate dilutions of the analytes stock solutions were made with methanol to prepare a standard solution for system suitability, at the concentration of 500 ng/ml.

The IS stock solution was diluted with 75% methanol/MTBE (9:1) to a concentration of 500 ng/ml and used as extraction solution. All solutions were stored at -20°C.

Sample Preparation

A total of $1x10^6$ cells were washed twice with PBS, placed in a 1.5 mL tube, and pelleted. Then, 500 µL of ice-cold 75% methanol/MTBE (9:1) containing 2-Deoxy-D-glucose 6-phosphate (IS 500 ng/mL) was added to each tube. Samples were briefly vortexed, and the cell suspension was lysed by

three freeze-thaw cycles (fresh frozen in liquid nitrogen and thawed at 37°C for 10 min under shaking). After cell disruption and centrifugation at 14000g for 10 min at 4°C, the supernatant was transferred to a new 1.5 mL Eppendorf tube, dried under nitrogen, and reconstituted with 0.1% FA in water. Extracts were filtered with a 0.22 µm RC syringe filter and 10 µL were injected onto the chromatographic system.

Statistical Analysis

Descriptive statistics were carried out with Prism GraphPad 8.0.1 (San Diego, CA, USA). All analytes' peak areas were normalized by IS area. A two-tailed parametric paired Student's t-test was used to assess significant differences between miR-22-modulated cells vs control (shRNA) cells. A p-value < 0.05 was considered significant. Fold changes were calculated as a ratio of the normalized peak area of miR-22-modulated cells to control cells.

IS Internal Standard
LC-MS Liquid chromatography – mass spectrometry
HPLC High-pressure liquid chromatography
UHPLC Ultra high-pressure liquid chromatography
MS/MS Tandem mass spectrometry
MRM Multiple reaction monitoring
MTBE *ter*-butil metil etere
RC Regenerated cellulose

References

- R Core Team (2020). — European Environment Agency Available from: https://www.eea.europa.eu/data-and-maps/indicators/oxygen-consuming-substances-in-rivers/r-development-core-team-2006. Accessed August 31, 2022
- Love MI, Huber W, Anders S. Moderated estimation of fold change and dispersion for RNA-seq data with DESeq2. Genome Biol 2014;15:550
- Korotkevich G, Sukhov V, Budin N, Shpak B, Artyomov MN, Sergushichev A. Fast gene set enrichment analysis. 2021;060012
- Subramanian A, Tamayo P, Mootha VK, Mukherjee S, Ebert BL, Gillette MA, Paulovich A, Pomeroy SL, Golub TR, Lander ES, Mesirov JP. Gene set enrichment analysis: A knowledge-based approach for interpreting genome-wide expression profiles. Proc Natl Acad Sci 2005;102:15545–15550
- Mootha VK, Lindgren CM, Eriksson K-F, Subramanian A, Sihag S, Lehar J, Puigserver P, Carlsson E, Ridderstråle M, Laurila E, Houstis N, Daly MJ, Patterson N, Mesirov JP, Golub TR, Tamayo P, Spiegelman B, Lander ES, Hirschhorn JN, Altshuler D, Groop LC. PGC-1alpha-responsive genes involved in oxidative phosphorylation are coordinately downregulated in human diabetes. Nat Genet 2003;34:267–273
- Liparulo I, Bergamini C, Bortolus M, Calonghi N, Gasparre G, Kurelac I, Masin L, Rizzardi N, Rugolo M, Wang W, Aleo SJ, Kiwan A, Torri C, Zanna C, Fato R. Coenzyme Q biosynthesis inhibition induces HIF-1α stabilization and metabolic switch toward glycolysis. FEBS J. 2021. 288, 1956–1974. https://doi.org/10.1111/febs.15561.
- Rizzardi, N., Liparulo, I., Antonelli, G., Orsini, F., Riva, A., Bergamini, C., Fato, R., Coenzyme Q10 Phytosome Formulation Improves CoQ10 Bioavailability and Mitochondrial Functionality in Cultured Cells. Antioxid. 2021.10, 927. https://doi.org/10.3390/antiox10060927.
- Righi V, Mucci A, Schenetti L, Tosi MR, Grigioni WF, Corti B, Bertaccini A, Franceschelli A, Sanguedolce F, Schiavina R, Martorana G, Tugnoli V. Ex vivo HR-MAS magnetic resonance spectroscopy of normal and malignant human renal tissues. Anticancer Res. 2007. 27:3195–3204
- Price WS, Hayamizu K, Ide H, Arata Y. Strategies for diagnosing and alleviating artifactual attenuation associated with large gradient pulses in PGSE NMR diffusion measurements. J Magn Reason. 1999. 139: 205–212
- Schenetti L, Mucci A, Parenti F, Cagnoli R, Righi V, Tosi MR, Tugnoli V. HR-MAS NMR spectroscopy in the characterization of human tissues: Application to healthy gastric mucosa. Concepts Magn. Reson. Part A. 2006. 28A, 430–443
