# Supplementary figures and images for: MiR-22/GLUT1 Axis Induces Metabolic Reprogramming and Sorafenib Resistance in Hepatocellular Carcinoma
Source: Int J Mol Sci. 2025 Apr 17;26(8):3808. doi: 10.3390/ijms26083808 (PMC12027541; doi:10.3390/ijms26083808)

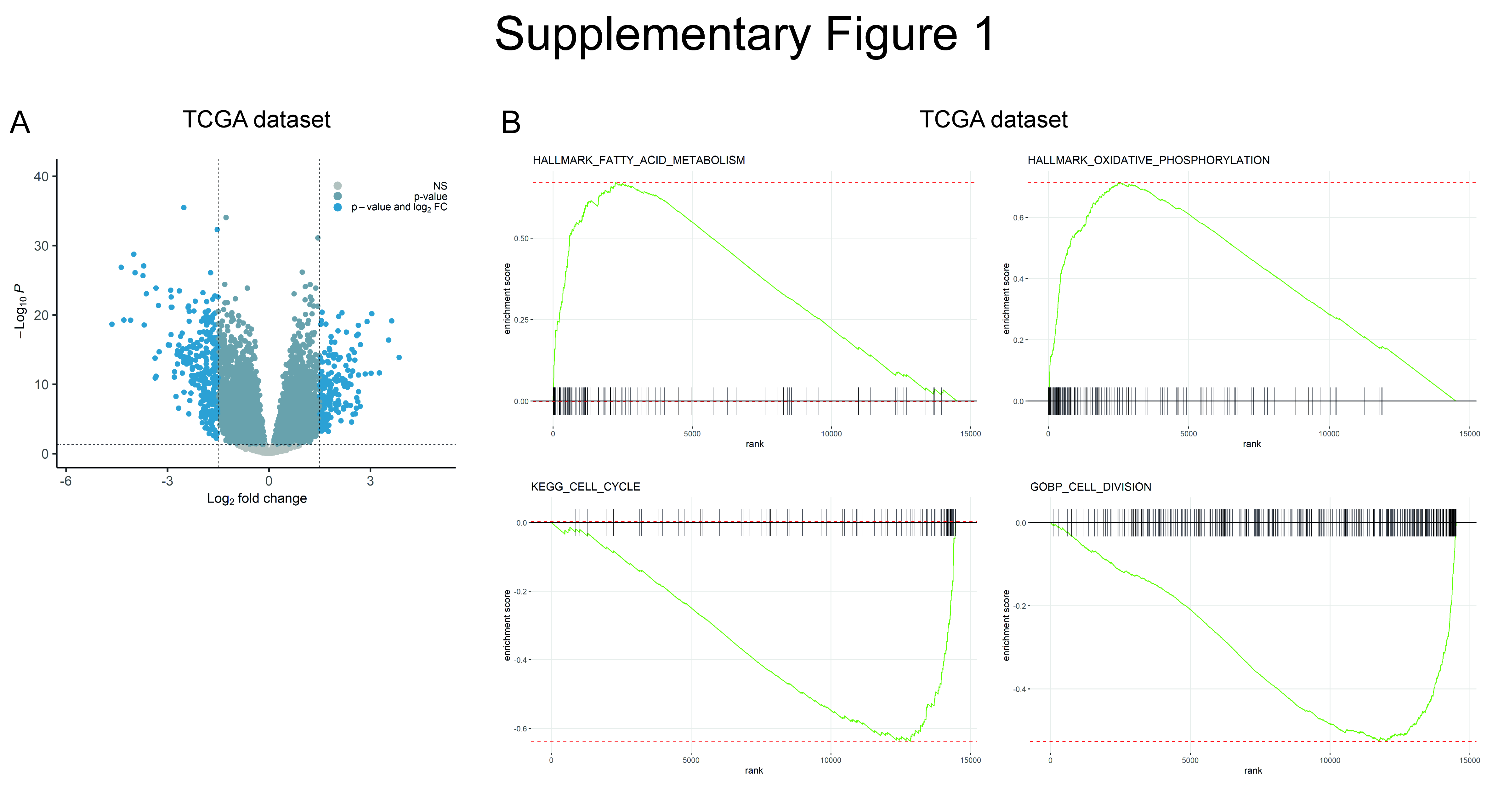

Supplement: Supplementary file 1 [file ijms-26-03808-s001.zip › Supplementary Figure S1.tif]

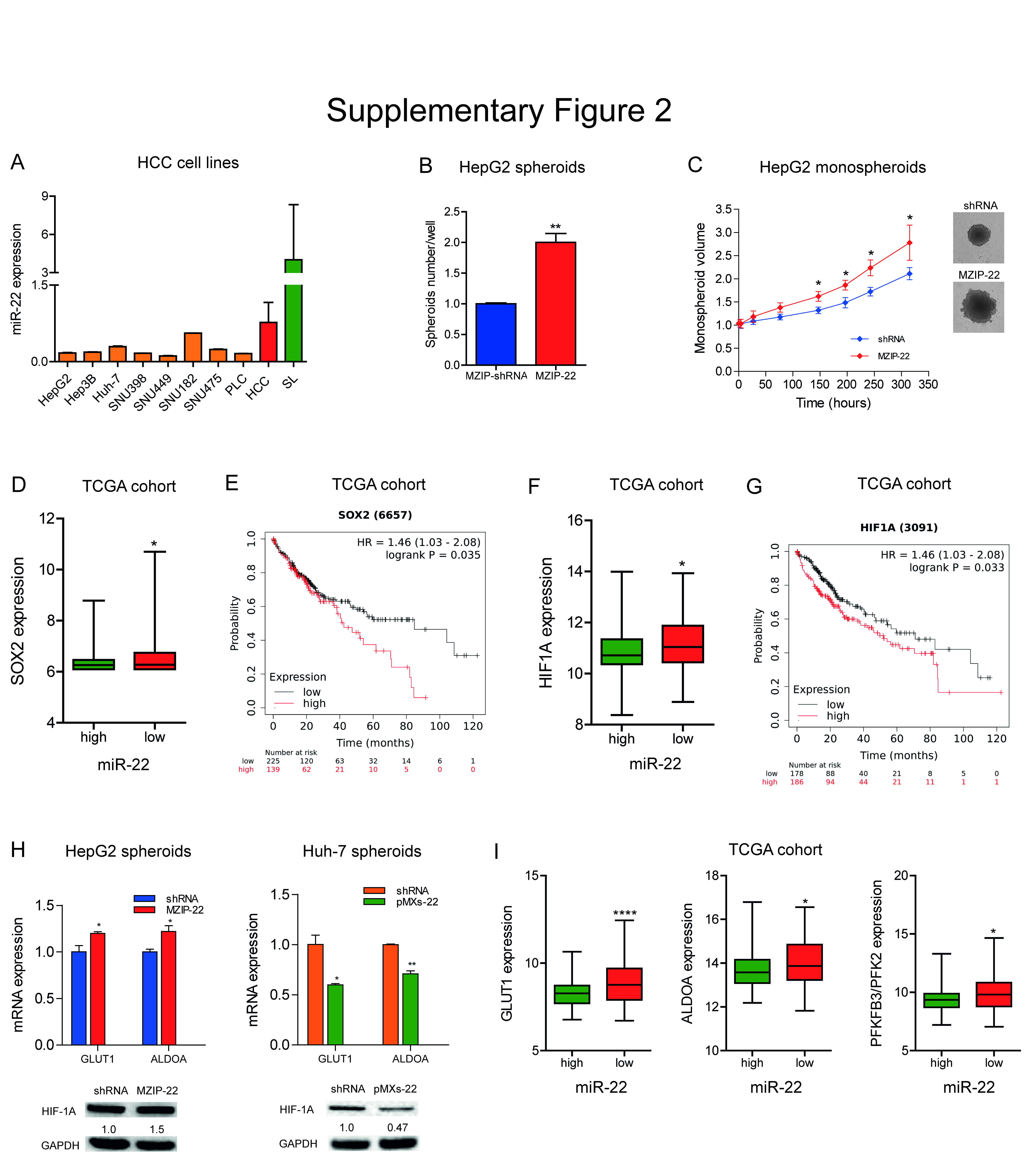

Supplement: Supplementary file 1 [file ijms-26-03808-s001.zip › Supplementary Figure S2.tif]

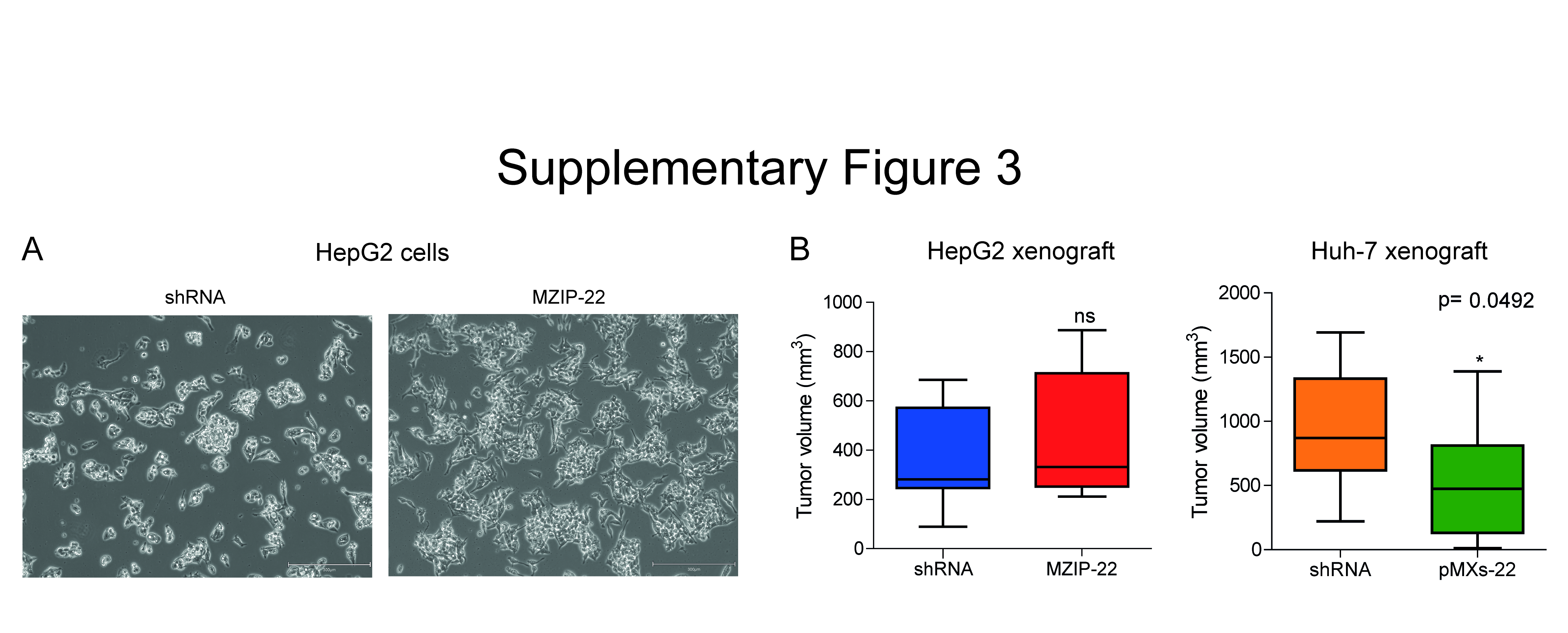

Supplement: Supplementary file 1 [file ijms-26-03808-s001.zip › Supplementary Figure S3 - EMT.tif]

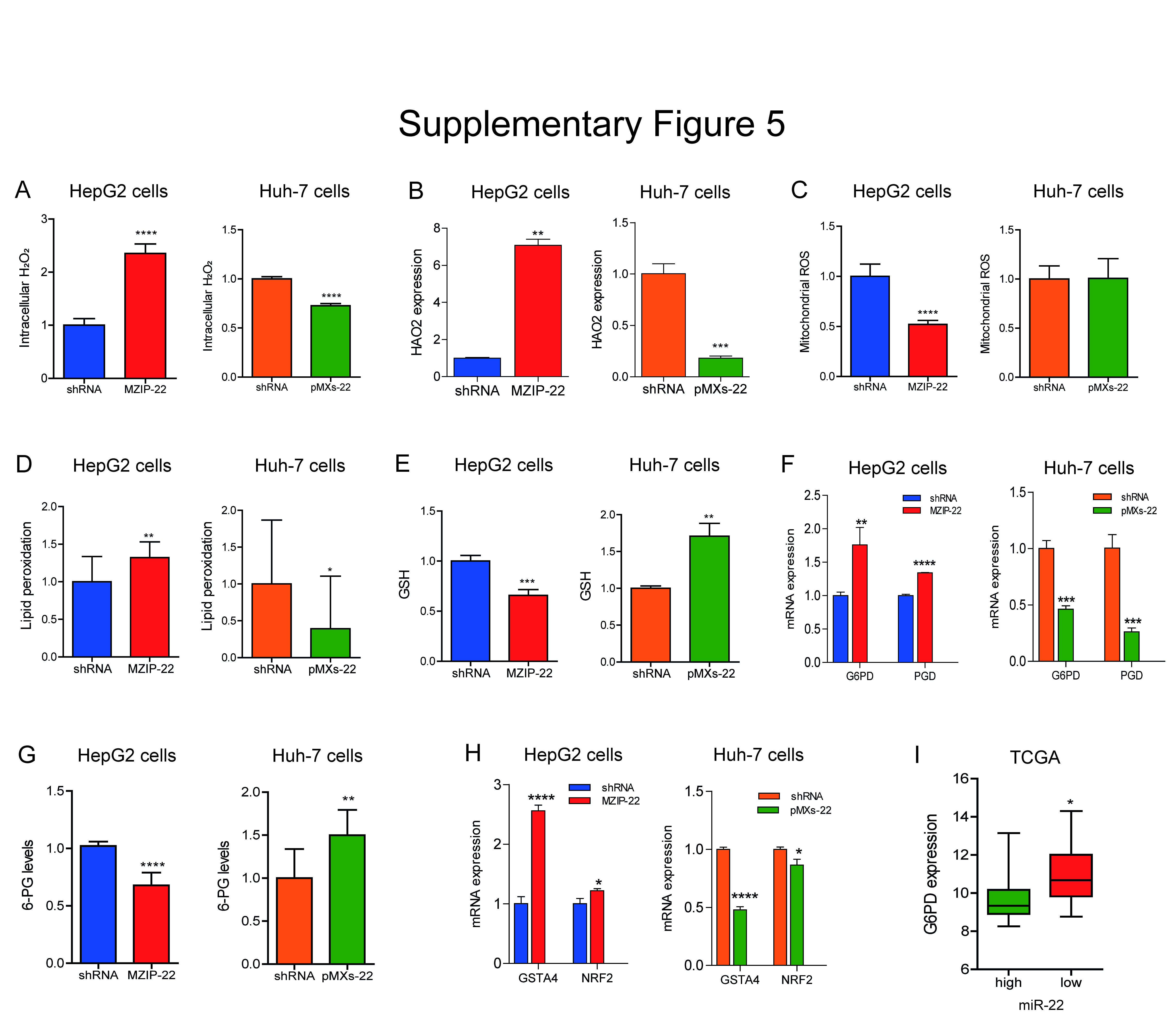

Supplement: Supplementary file 1 [file ijms-26-03808-s001.zip › Supplementary Figure S5 - ROS.tif]
